# Epigallocatechin gallate-zinc oxide co-crystalline nanoparticles as an anticancer drug that is non-toxic to normal cells†

**DOI:** 10.1039/c7ra10997k

**Published:** 2018-02-15

**Authors:** Pawatsanai Samutprasert, Khajeelak Chiablaem, Chanon Teeraseranee, Punnawich Phaiyarin, Puttikorn Pukfukdee, Prompong Pienpinijtham, Jisnuson Svasti, Tanapat Palaga, Kriengsak Lirdprapamongkol, Supason Wanichwecharungruang

**Affiliations:** Department of Chemistry, Faculty of Science, Chulalongkorn University Thailand psupason@chula.ac.th; Center of Excellence on Petrochemical and Materials Technology, Chulalongkorn University Bangkok 10330 Thailand; Laboratory of Biochemistry, Chulabhorn Research Institute Bangkok 10210 Thailand; Department of Nanoengineering, Faculty of Engineering, Chulalongkorn University Thailand; Department of Microbiology, Faculty of Science, Chulalongkorn University Thailand; Center of Excellence on Materials and Bio-interfaces, Chulalongkorn University Bangkok 10330 Thailand

## Abstract

Decreased uptake and cellular accumulation of zinc is a common characteristic in cancer of the liver, pancreas and prostate, because these malignant cells are intolerant to the physiological concentrations of zinc. A tea polyphenol, epigallocatechin-3-gallate (EGCG), can enhance the cytotoxicity of zinc ions to cancer, but the application of this is limited by the low stability of EGCG. In this work, we have prepared a material that can simultaneously preserve the EGCG stability and facilitate zinc uptake and accumulation in cancer cells, under conditions that are not harmful to normal cells. Thus, we co-crystallize zinc oxide with EGCG to obtain hybrid EGCG-ZnO crystalline nanoparticles of 16.5 ± 5.3 nm in diameter. The EGCG-ZnO particles effectively kill PC-3 prostate adenocarcinoma cells at concentrations that are not cytotoxic to normal cells, WI-38 human embryonic lung fibroblasts. The EGCG-ZnO particles are two times more cytotoxic against PC-3 cells than the standard ZnO particles. In PC-3 cells, the EGCG-ZnO particles are taken up by endocytosis, followed by lysosomal disruption to release zinc and EGCG into the cytoplasm, finally resulting in nuclear accumulation of zinc.

## Abstract 2

A tea polyphenol, epigallocatechin-3-gallate (EGCG), can enhance cytotoxicity of Zinc in cancer cells. Here we synthesize hybrid EGCG-ZnO nanoparticles that can kill PC-3 prostate cancer cells at concentrations that are not toxic to normal cells.

## Graphical abstract

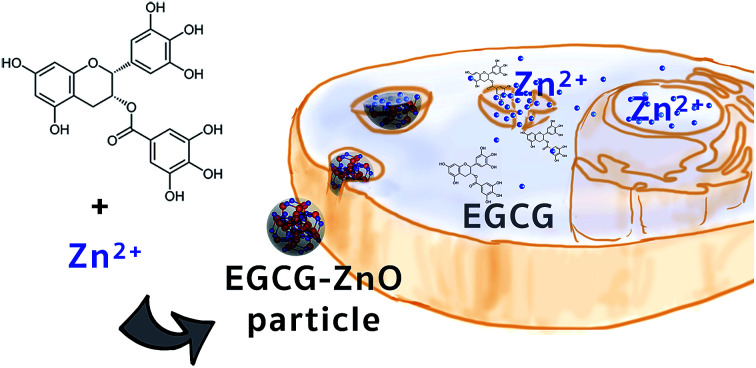

## Introduction

Zinc is the most abundant trace element in the human body and usually exists in the Zn^2+^ state. The estimated cellular concentration of zinc is ∼100–800 μM, with approximately 90% being tightly complexed with various proteins and the other 10% being chelated by small nucleophilic molecules such as citrate, aspartate, cysteine and histidine.^1^ Zinc is known to be involved in various cellular processes such as DNA synthesis, apoptosis, gene expression, protein stability and catalytic functions.^2^ Although it is a common metal ion in the human body, zinc also possesses anticancer activity.^3^ Zinc can restrict the growth of prostate cancer cells *via* cell cycle arrest, as well as induction of apoptosis and necrosis.^4,5^ Several lines of evidence indicate that downregulation of ZIP-family zinc transporter and decrease in intracellular zinc levels is commonly observed in the development and progression of prostate, liver, and pancreatic cancers.^6^ This phenomenon is termed ZIP/Zn transformation and has been thought to protect the cancer cells from cytotoxic effect of the zinc levels found in normal cells. Therefore, any strategies that can increase zinc levels in the cancer cells are likely to be effective cancer treatment tactics having minimal toxicity to normal cells. Accordingly, various complexes between Zn^2+^ and bioactive molecules have been prepared and explored in the development of new antitumor drugs.^7–9^

ZnO is a semiconductor that has been proposed to be photo or sono-sensitizer based cancer therapeutic material.^10^ However, ZnO can also be used for cancer treatment in the absence of light, *via* its ability to release Zn^2+^ upon the dissolution under mild acidic conditions. As with the effects of zinc on normal and cancer cells, cytotoxicity of ZnO is more pronounced in rapidly dividing cells, such as tumor cells, than in quiescent cells or normal cells.^11^ This has encouraged researchers to use ZnO as a pH responsive depot for Zn^2+^, *e.g.* the design of ZnO quantum dots that were stable at physiological pH but readily disintegrated into Zn^2+^ in the mildly acidic environment of cancer cells.^12^ ZnO-polymer particles have been designed to release Zn^2+^ when exposed to acidic conditions within endosomes after being taken up by the cells *via* endocytosis.^13^ Nevertheless, ZnO is not a completely safe material; Han *et al.* reported the unwanted effects of ZnO nanoparticles injected into male mice leading to structural alterations in the seminiferous epithelium and sperm abnormalities.^14^

Epigallocatechin-3-gallate (EGCG), the major polyphenolic compound found in tea leaves, exhibited the growth inhibitory effect on cancer cells *in vitro*, in animal tumor models and in clinical trials.^15–18^ EGCG exerts anticancer activity by modulation of multiple processes, including proliferation, migration and invasion of cancer cells, as well as promoting cancer cell death, and interrupting the action of various carcinogens, thus preventing tumorigenesis. Nevertheless, application of EGCG for cancer treatment faces obstacles due to low stability, low bioavailability, poor absorption and fast elimination of EGCG.^19,20^

Synergistic action of zinc and EGCG in cancer treatment has been observed in various cell lines,^21,22^ but the mechanism for this is still unclear. Hagerman *et al.* (2003),^23^ have reported that EGCG radicals can be formed easily at low pH, and these radicals can be spin-stabilized by Zn^2+^ The Zn^2+^–EGCG radical complex formed can more effectively damage proteins, compared to unoxidized EGCG molecules. Recently, Dabbagh-Bazarbachi *et al.* (2014),^24^ have demonstrated that EGCG can act as an ionophore that helps to transport zinc across cell membrane. The authors also proposed that the zinc ionophore activity of EGCG might cause the disruption of mitochondria function in the presence of zinc, and thus exert cytotoxicity.

This paper demonstrates a strategy for facilitating the uptake and accumulation of zinc in cancer cells while simultaneously preserving EGCG stability and using the EGCG to improve the effectiveness of zinc in the killing of cancer cells, under conditions that are not toxic to normal cells. We have co-crystalized zinc oxide with EGCG to obtain the hybrid EGCG-ZnO nanoparticles, *via* hydrothermal synthesis. The obtained particles were chemically characterized, and their cytotoxicity against normal macrophage cells and prostate cancer cells was evaluated. Endosomal disruption ability and intracellular trafficking of the EGCG-ZnO hybrid particles and intracellular localization of released Zn^2+^ were monitored in live cells.

## Experimental section

### EGCG extraction

Dried Oolong tea leaves (26 g, Jin Xuang No. 12, Boonrod Farm, Chiang Mai, Thailand) were stirred with methanol (300 mL) at 55 °C for 3 h, then the mixture was cooled, and solid residues were filtered out. The liquid extract was evaporated under the reduced pressure at 50 °C to obtain the crude extract. The crude extract was partitioned with 40 mL of dichloromethane : methanol : water (3 : 1 : 0.2 v/v) mixture. The methanol/water layer was collected and evaporated under low pressure at 55 °C to obtain the extracted EGCG (5.04 g). The obtained EGCG was subjected to ^1^H NMR (DMSO-D_6_, 400 MHz Varian Mercury^+^ NMR spectrometer) and reverse phase HPLC analyses.

### Preparation of EGCG-ZnO hybrid nanoparticles

To prepare EGCG-ZnO hybrid nanoparticles, zinc chloride (873 mg, Merck, kGaA, Germany) and EGCG (27 mg) were dissolved in water (50 mL), and 1.25 M sodium hydroxide solution was added until the pH of the mixture reached 7.3. The mixture obtained was then refluxed at 100 °C for 3 h. The suspension was cooled and placed at ambient temperature for 3 days before being centrifuged (15 000 rpm for 15 min), and the pellet obtained was collected and washed with water. Supernatant from the centrifugation process was subjected to HPLC analysis to quantify for organic entities left in the solution. Finally, the pale-yellow product was subjected to scanning electron microscopy (SEM, JEOL, JSM-7610), transmission electron microscopy (TEM, JEOL JEM-2100), X-ray diffraction (XRD, Rigaku D/MAX-2200 Ultima-plus with Cu Kα, *λ* = 1.5418 A, 40 kV, mA), ATR-FTIR (Nicolet 6700 ATR-FT-IR spectrometer, Thermo Electron Corporation), thermogravimetric (TGA, Netzsch STA 449 F1), and X-ray photoelectron spectroscopy (XPS, a Kratos AXIS Ultra DLD) analyses. The standard ZnO and the obtained particles were separately dispersed in water and each suspension was subjected to dynamic light scattering analysis (Malvern Zetasizer nanoseries model S4700, He–Ne laser beam at 632.8 nm, scattering angle of 173°) to obtain the hydrodynamic sizes and zeta potential values.

### HPLC analysis

HPLC analysis of the extracted EGCG and the supernatant from ZnO preparation process was carried out using the Waters 1525 binary HPLC pump coupled to an UV-visible detector (set at 270 nm, Waters 2489) and Hypersil C18 column (100 × 4.6 mm i.d., Thermo Fisher Scientific Inc, Waltham, MA, USA). The flow rate was 0.7 mL min^−1^, injection volume was 22 μL, and the sample was dissolved in acetonitrile at 100 mg mL^−1^. The gradient elution started with 95% of 0.1% aqueous formic acid and 5% acetonitrile. Then, the content of acetonitrile was increased linearly to 15% within 14 min and maintained for 11 min. After that, the percentage of acetonitrile was increased to 35% within 28 min, and to 85% within 12 min. Analysis was carried out in triplicate. Series of solutions of each standard were analyzed under the same HPLC conditions to establish the calibration curves. The standard solutions include 30–70 μg mL^−1^ gallic acid, 150–800 μg mL^−1^ caffeine, 120–500 μg mL^−1^ epigallocatechin-3-gallate (EGCG) and 40–120 μg mL^−1^ epicatechin-3-gallate (all polyphenols were at least 90% pure and were purchased from Chemieliva Pharmaceutical Co., Ltd., Chongqing, China. Each standard was analyzed by ^1^H NMR).

### Extraction of organic materials from the hybrid nanoparticles

The EGCG-ZnO nanoparticles were added to aqueous 5 M hydrochloric acid solution to give a final concentration of 200 μg mL^−1^. Then the clear liquid was immediately subjected to EGCG analysis by UV-vis absorption spectrophotometry.

### Doxorubicin loading

The EGCG-ZnO particles were dispersed in water and doxorubicin (DOX, purchased from Sigma-Aldrich Chemical Co., St. Louis, MO, USA) solution was added to the suspension (final concentrations of EGCG-ZnO particles and DOX were 2000 and 200 μg mL^−1^, respectively). The mixture was incubated at 30 °C in the dark for 24 h. The mixture was then centrifuged (12 225 × g, 10 min) and the pellet was collected. The supernatant was quantified for unloaded DOX by UV-visible spectroscopy with the aid of the calibration curve. The encapsulation efficiency (% EE) of the process and the loading capacity (% loading) of the particles were obtained as follows:1
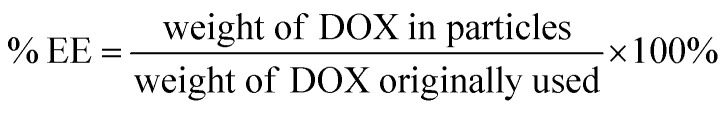
2
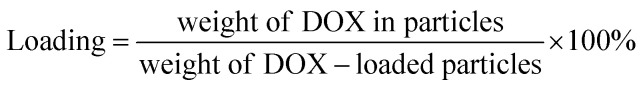


### Particle disintegration monitoring

To monitor the dissolution of EGCG-ZnO particles (or standard ZnO particles, Sigma-Aldrich), the aqueous suspensions of each tested sample were prepared to have the pH of 4.5 to 7.0, all at a final concentration of 150 μg mL^−1^. Light transmission through each suspension was measured using red light (630 nm) using the Ocean Optics USB4000 Fiber Optic Spectrometer coupled with a DH-2000 halogen light source (Mikropack, Dunedin, USA).

### Cytotoxicity determination in normal and cancer cells

WI-38 human embryonic lung fibroblasts and PC-3 human prostate adenocarcinoma cell line (American Type Culture Collection) were cultivated in DMEM and F-12K media, respectively. The media were supplemented with 10% (v/v) FBS, 100 U mL^−1^ penicillin, 100 μg mL^−1^ streptomycin and 125 ng mL^−1^ amphotericin B, at 37 °C in humidified atmosphere of 5% CO_2_.

Cell viability was measured by our previously used MTT assay with some modification.^25^ Briefly, cell suspensions in completed media were seeded into a 96-well plate at 1 × 10^4^ cells per well and incubated at 37 °C in humidified atmosphere of 5% CO_2_. After 24 h, additional media (100 μL) containing the tested sample were added to each well, followed by further incubation for 72 h. Then, the wells were replaced and incubated with fresh media containing 0.5 mg mL^−1^ MTT, for 2 h at 37 °C. Finally, the media were removed and 100 μL of DMSO were added to each well. The absorbance was measured at 550 nm and subtracted with absorbance at 650 nm, using a microplate reader. The number of viable cells was determined from the absorbance.

### Intracellular trafficking

The PC-3 cells were seeded into an 8-well chamber slide, at 1 × 10^4^ cells/200 μL per well and allowed to adhere overnight. CellLight™ Early Endosome-RFP BacMam 2.0 reagent (*λ*_excite_/*λ*_emit_ of 559/584 nm, Molecular probes, Life technologies, Carlsbad, CA, USA) was added into each well (2 μL/10 000 cells) and incubated at 37 °C in 5% CO_2_ for 16 h. Then, 75 μL of 2 μM lysotracker deep red (*λ*_excite_/*λ*_emit_ of 635/658 nm, molecular probes) in 0.1 mM phosphate buffer saline (PBS) and 3 μL of 30 μM zinquin ester solution (a Zn^2+^-specific fluorescent probe, *λ*_excite_/*λ*_emit_ of 405/450 nm, Sigma-Aldrich) in DMSO, were added to each well and incubated for another 20 min, before the addition of the suspension of doxorubicin-loaded-EGCG-ZnO (100 μL, 9.7 μg mL^−1^ EGCG, 69.7 μg mL^−1^ doxorubicin *λ*_excite_/*λ*_emit_ of 473/570 nm, and 920.6 μg mL^−1^ ZnO) to each well. After that the live cells were immediately monitored under confocal fluorescence microscope (Olympus, Fluoview, FV10i-LIV) at 37 °C in a CO_2_ incubator. The image data were analyzed through FV10-ASW software. Image indicating locations of each emission channel was then constructed. Similar experiment was carried out with EGCG-ZnO particles (100 μL, 9.7 μg mL^−1^ EGCG, 920.6 μg mL^−1^ ZnO).

## Results and discussion

By soaking the tea leaves in methanol followed with dichloromethane partition, tea polyphenols could be extracted from the Oolong tea leaves with the yield of 23% (dry extract wt to dry tea leaf wt) HPLC analysis as referenced to retention times of standard compounds indicated EGCG as the major constituent of the extract obtain (Fig. S1†). Other minor constituents of the extract include caffeine, epicatechin-3-gallate and gallic acid. ^1^H NMR analysis of the extract also confirmed that EGCG was the major constituent (Fig. S2 and S3†). The water-soluble extract obtained having predominant EGCG was used in the preparation of the hybrid ZnO particles.

It is known that ZnO particles can be prepared from zinc ion precursors such as zinc chloride, zinc acetate and zinc nitrate, under basic pH conditions, at low to moderate temperature in water medium. This method is called a hydrothermal or solvothermal process, and usually yields ZnO with high crystallinity and high purity. In this method, the morphology and size of the synthesized particles are affected by various factors such as reaction temperature, concentration of base, time and the presence of various additives.^26–29^ Among known materials, ZnO has been prepared in numerous morphological varieties,^30^ including nanorods/wires,^31^ needles,^32^ helixes, springs and rings,^33^ belts,^34^ and flower.^35^

**Fig. 1:**
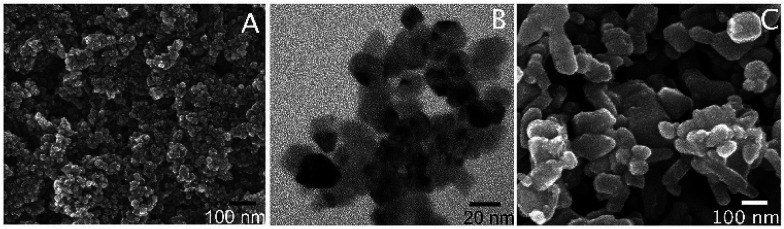
SEM (A) and TEM (B) of the hybrid EGCG-ZnO nanoparticles, and SEM image (C) of standard ZnO particles.

**Fig. 2:**
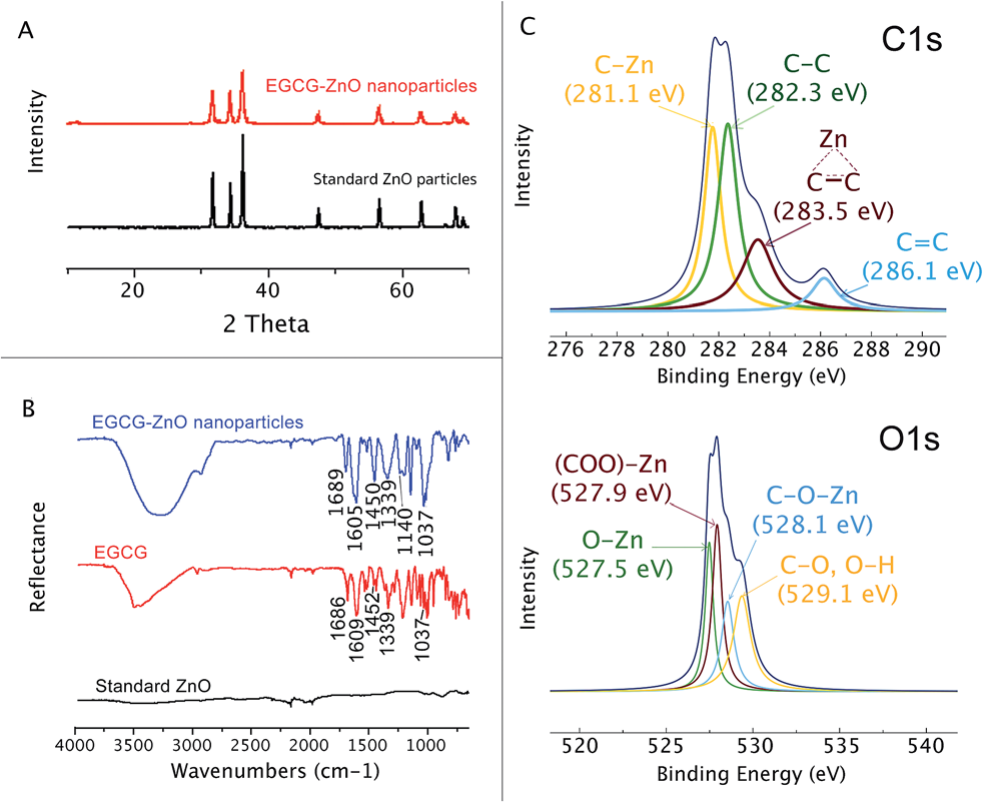
Characterization of EGCG-ZnO. (A) X-ray diffraction patterns of EGCG-ZnO nanoparticles and standard ZnO crystal. (B) FT-IR spectra of EGCG-ZnO nanoparticles, EGCG and standard zinc oxide crystal. (C) X-ray photoelectronic spectra of EGCG-ZnO nanoparticles.

Here we used the EGCG as an additive to control the size and shape of ZnO particle during the solvothermal preparation from Zn^2+^. By adjusting the pH of the aqueous zinc chloride solution to 7.3 in the presence of the extracted EGCG, and then refluxing the mixture for 3 h, a colloid was formed. The particles formed could be isolated from the solution by centrifugation. Organic matters left in the solution were identified and quantified by HPLC. The analysis revealed that EGCG was the main organic matter in the isolated colloid and accounted for 1.1% by weight of the hybrid EGCG-ZnO particles. SEM and TEM analyses indicated spherical morphology with an average diameter of approximately 16.5 ± 5.3 nm (Fig. 1A and B). The obtained EGCG-ZnO particles are smaller and more uniform than the commercially available standard ZnO particles (Fig. 1C). Interestingly, both particles show much larger hydrodynamic diameters in water, 409.5 ± 9.2 nm for EGCG-ZnO and 297.5 ± 4.5 nm for standard ZnO. Aggregation of the particles into larger entities probably occurred, and this conformed with their zeta potential values of −20.03 ± 0.23 and −25.00 ± 1.74 mV for EGCG-ZnO and standard ZnO, respectively (Table S1†). The XRD pattern of the obtained nanoparticles resembles the pattern of the standard zinc oxide crystal (Fig. 2A), thus confirming the hexagonal wurtzite crystal structure of the prepared nanoparticles. The FT-IR spectrum of the obtained nanoparticles (Fig. 2B) showed the characteristic peaks of polyphenols, *e.g.*, C=C stretching at 1588.48 cm^−1^ (benzene ring), O–H stretching at 1384.02 cm^−1^ (hydroxyl group) and C–O stretching at 1043.24 cm^−1^. Similarity between the FT-IR spectrum of the hybrid particles and the spectrum of standard EGCG (98% purity) implied that EGCG is the main organic constituent in the hybrid particles, agreeing well with the above HPLC analysis. Therefore, we conclude that the obtained particles were hybrid particles of crystalline ZnO and EGCG.

Functional groups on the surface of the EGCG-ZnO nanoparticles were estimated through its X-ray photoelectronic spectra (Fig. 2C). The deconvoluted C 1s XPS spectrum of the EGCG-ZnO nanoparticles shows the four binding energies peaks at 281.1 eV, 282.3 eV, 283.5 eV and 286.1 eV, corresponding to C–Zn, C–C sp^3^, C–C sp^2^ and C=C, respectively. The deconvoluted O 1s XPS spectrum of the EGCG-ZnO particles exhibits the binding energy at 527.5 eV (O–Zn), 527.9 eV (O=C–O–Zn from carboxyl group) and 529.1 (C–O, OH). The presence of the hydrophilic hydroxyl and carbonyl groups at the surfaces of the particles agrees well with their good water dispersability. The existence of C=C and C=O functionalities on the surface of the particles conforms well to the presence of polyphenol in the particles.

We monitored the solubilisation (disintegration) of the obtained hybrid EGCG-ZnO particles and standard ZnO particles at various pH conditions using a light scattering technique. The particles were dispersed in aqueous media of various pH values and allowed to incubate for 3 min before subjecting to light scattering measurement. Partial solubilisation of the two particles was observed at pH 5.5, and complete solubilisation was observed at pH ≈ 4.5 (Fig. S4†). This means that both the hybrid EGCG-ZnO nanoparticles and the standard ZnO particles are acid-responsive regarding the disintegration/solubilisation of the particles.

**Fig. 3:**
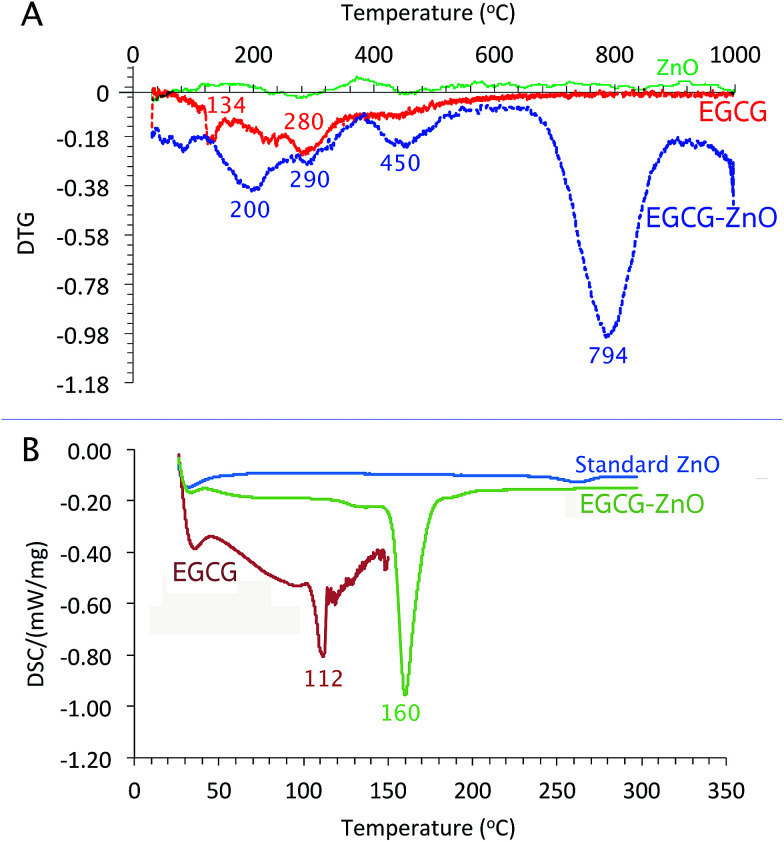
Thermal analyses of EGCG-ZnO: (A) differential thermos-gravimetric curve of EGCG, EGCG-ZnO and standard ZnO, (B) differential scanning calorimetric thermograms of EGCG, EGCG-ZnO and standard ZnO.

As shown in Fig. 3A, the differential thermos-gravimetric curve of the extracted EGCG shows the broad characteristic weight loss at 120–400 °C with obvious peak maxima at 134 and 280 °C, implying that the material degrades at 120–400 °C. Standard ZnO shows insignificant weight loss at 0–1000 °C. Interestingly, the differential thermo-gravimetric curve of EGCG-ZnO shows the biggest weight loss at 794 °C and smaller weight loss around 120–500 °C with the maxima at 200, 290 and 450 °C, corresponding well to the presence of organic matters in the particles. The differences in thermal mass loss characteristics between the EGCG and the EGCG-ZnO indicate strong interaction of the polyphenol molecules with ZnO crystal.^36^

As shown in Fig. 3B, the usual characteristic endothermic absorption peak of the extracted EGCG at 112 °C could not be observed in the differential scanning calorimetric (DSC) profile of the hybrid particles, but the endothermic peaks at 160 °C was observed instead. This indicates interaction between the EGCG molecules and the ZnO crystals in the hybrid nanoparticles.

**Fig. 4:**
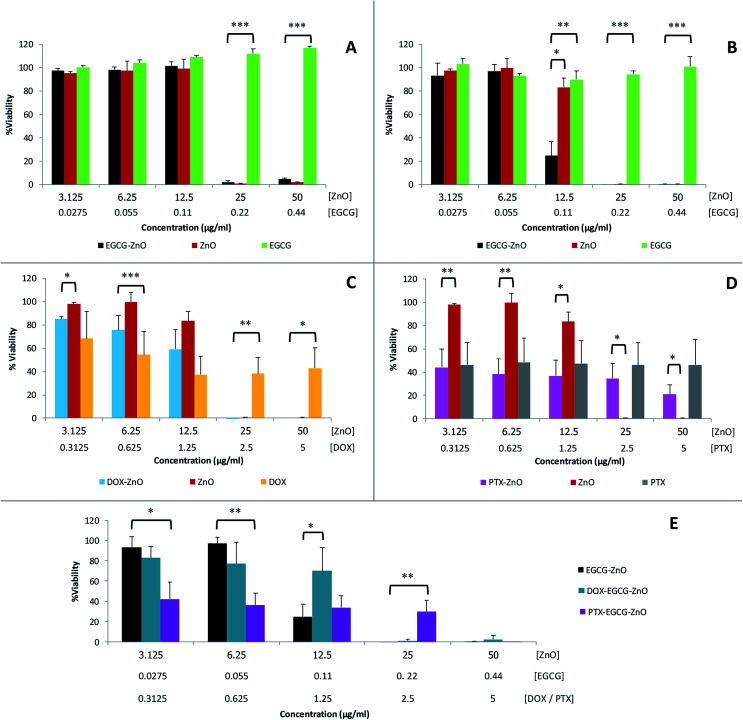
Cytotoxicity of various materials in normal cells and cancer cells as measured by MTT assay: EGCG-ZnO particles, standard ZnO particles and EGCG, to WI38 cells (A) and PC-3 cells (B); DOX-ZnO, standard ZnO, and DOX to PC-3 cells (C); PTX-ZnO, standard ZnO, and PTX to PC-3 cells (D); EGCG-ZnO particles, DOX-loaded EGCG-ZnO particles, PTX-loaded EGCG-ZnO particles, and free DOX and free PTX, in PC-3 cells (E). Data are expressed as mean ± S.D. of 3 independent experiments. Statistical difference was analyzed by Student's *t*-test; *P < 0.05, **P < 0.01, and **P < 0.001 were considered statistically significance.

We further evaluated the cytotoxicity of the extracted EGCG, standard ZnO and the hybrid EGCG-ZnO particles in normal fibroblast cells (WI-38) and prostate cancer cells (PC-3), using MTT assay. EGCG alone, at the tested concentrations of up to 0.44 μg mL^−1^, affect viability of neither normal cells nor cancer cells (Fig. 4A and B, green bars). In contrast, standard ZnO particles at 25–50 μg mL^−1^ produced nearly 100% cell death in both normal and cancer cells (Fig. 4A and B, red brown bars). As expected, cancer cells were more sensitive to hybrid EGCG-ZnO particles than the normal cells. At 12.5 μg mL^−1^, the hybrid EGCG-ZnO particles produced 75% cell death in cancer cells, whereas viability of normal cells was not affected (Fig. 4A and B, black bars). Comparing to the standard ZnO particles, the results indicated that the EGCG-ZnO particles were more cytotoxic to cancer cells but less toxic to normal cells, IC_50_ values of EGCG-ZnO particles in cancer cells were 2-fold lower than the IC_50_ values in normal cells (see Table S2†). This property suggests that the hybrid EGCG-ZnO particles possess a cancer-selective potential. In other words, EGCG-ZnO particles can effectively kill prostate cancer cells at a concentration that is not toxic to normal cells.

The above results are in accord with our hypothesis that the presence of small amounts of EGCG in hybrid EGCG-ZnO in cancer cells and normal cells, the results were identical to that of standard ZnO alone (data not show). Therefore, an explanation might be that the hybrid particles allow simultaneous uptake of EGCG and ZnO into cancer cells, and upon dissolution of the hybrid particles, the resulting Zn^2+^ and EGCG are both released into cytoplasm at the same time, so they can act synergistically inside the cells. In contrast, EGCG and ZnO in the mixture might be taken up separately by the cancer cells, and due to their differences in cellular penetrating abilities, penetration pathway and intracellular localization, they cannot act synergistically. To investigate whether ZnO could synergize with other anticancer drugs in addition to EGCG, we prepared doxorubicin-ZnO (DOX-ZnO) particles and paclitaxel-ZnO (PTX-ZnO) particles, and evaluated their anticancer activities in PC-3 prostate cancer cells. The results showed no synergistic action between these two anticancer drugs and ZnO in cancer cells (Fig. 4C and D). We further explored the use of EGCG-ZnO particles as a drug carrier to enhance delivery of DOX or PTX into cancer cells. Again, the results clearly showed no synergistic anticancer activity between the EGCG-ZnO carriers and the loaded drugs (Fig. 4E). In other words, the presence of neither DOX nor PTX enhanced cytotoxicity of the EGCG-ZnO particles.

Since the hydrodynamic size of EGCG-ZnO is only a little larger than that of the standard ZnO, it is unlikely that the higher cancer selective toxicity of EGCG-ZnO over that of the standard ZnO would be related to their larger wet size. It is likely that the synergism between EGCG and ZnO in the hybrid particles occurs selectively only in cancer cells. Moreover, EGCG and ZnO generally show low toxicity in normal cells. Taken together, these properties clearly make the hybrid EGCG-ZnO particles an interesting cancer-selective killing agent.

**Fig. 5:**
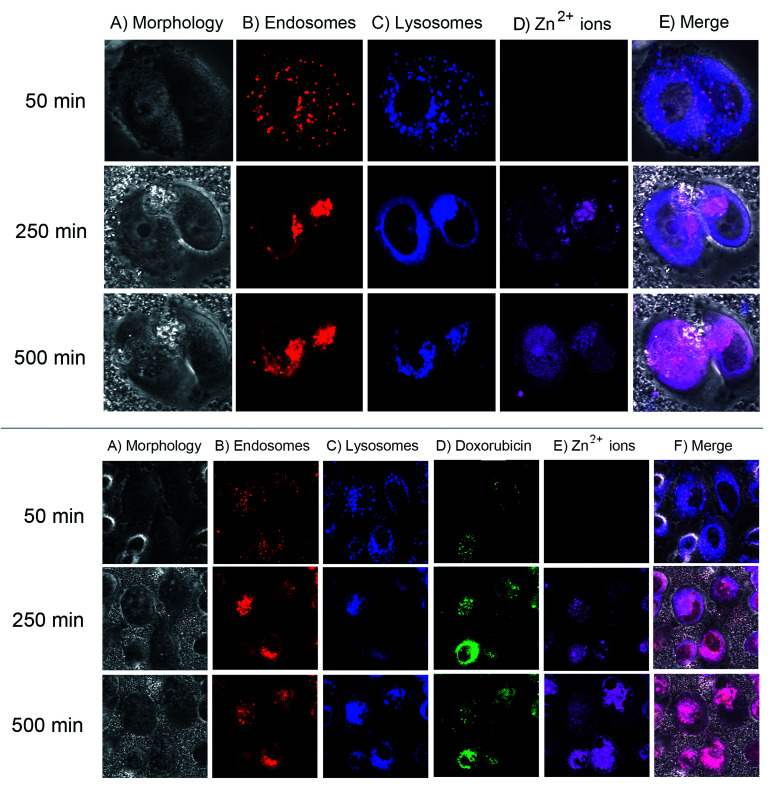
Intracellular trafficking of EGCG-ZnO hybrid nanoparticles (top) and DOX-loaded EGCG-ZnO particles (bottom) in PC-3 prostate cancer cells. Top: pictures showing cell morphology (column A), early endosome tracker signals (column B), lysosome tracker signals (column C), zinquin (column D) and the merged fluorescent signals (column E), at 50 min (row 1), 250 min (row 2) and 500 min (row 3) post-adding of the EGCG-ZnO particles into the cells. Bottom: pictures showing cell morphology (column A), early endosome tracker signals (column B), lysosome tracker signals (column C), doxorubicin (column D), zinquin (column E) and the merged fluorescence signals (column F), at 50 min (row 1), 250 min (row 2) and 500 min (row 3) post-adding of the Dox-loaded EGCG-ZnO particles into the cells.

We next monitored the cellular uptake and intracellular trafficking of the EGCG-ZnO particles in the prostate cancer cells. Disintegration of EGCG-ZnO particles was monitored by detecting the Zn^2+^ ions with zinquin, a fluorescent probe for Zn^2+^ ion detection.^37,38^ Endosomal and lysosomal compartments were also tracked by using early endosome specific dye and lysotracker deep red. However, in our experimental conditions, there was an overlapping of the fluorescent signals of both compartments, thus these signals were interpreted as location of endosomal/lysosomal compartments. As shown in (Fig. 5, top), when first appeared at 250 min post-treatment, the zinquin fluorescent signals co-localized with endosomal/lysosomal compartment fluorescent signals, implying that the EGCG-ZnO particles were taken up into cells *via* endocytosis and they disintegrated into Zn^2+^ inside the endosomal/lysosomal compartments. After that, at 500 min post-treatment, the zinquin signals were observed in cytoplasm and nucleus. The zinquin signals did not co-localize with the signals from endosomal/lysosomal compartments, indicating that Zn^2+^ leaked out from the compartments. We conclude that the EGCG-ZnO particles entered cells *via* endocytosis and disintegrated inside the endosomal/lysosomal compartments due to the reduction of pH inside the organelles, after which the zinc ions quickly translocate into the cytoplasm and accumulate in the nucleus. This result agreed well with our solubilisation experiment in which the hybrid EGCG-ZnO disintegrated slowly at pH 5.5 and dissolved quickly at pH 4.5 (Fig. S4†). The pH range of 5.5 (inside the endosome compartment) to 4.7 (inside the lysosome compartment) is low enough to trigger disintegration of the particles. The observation that Zn^2+^ accumulates in the nucleus implies that, in our cellular model, the anticancer effect of Zn^2+^ in the presence of EGCG occurs in the nucleus. Dipeptide and tetrapeptide complexes of Zn(ii), (Zn(ii)–Ala–Pro; Zn(ii)–Val–Pro; Zn(ii)–Pro–Gly–Pro–Gly) have been shown to hydrolyze phosphodiester bond at faster rates compared to the free ion, due to the participation of amino acid residues.^7^ In this work the authors also elucidated specific binding of the Zn(ii)–Pro–Gly to DNA. As for the Zn(ii) and EGCG, it was possible that the Zn(ii)–EGCG complex might form and might bind effectively to DNA in the nucleus, and efficient DNA cleavage then could take place with the hydrolytic catalysis role of Zn(ii). Various complexes of Zn^2+^ and small organic molecules have also been developed for anticancer purposes.^8,9^ In addition, studies have shown that in the presence of transition metal ions, *e.g.* copper, EGCG can exhibit pro-oxidant activity. Hayakawa *et al.* have reported a systematic study of *in vitro* DNA cleavage activity of EGCG and other catechins with various metal ions including Zn^2+^ and Cu^2+^.^39^ Later on, Azam *et al.* clearly demonstrated evidence for hydroxyl radical generation by EGCG in the presence of Cu^2+^ ions, which could induce DNA cleavage *in vitro*.^40^ Furthermore, another study showed a relationship between EGCG and Cu^2+^ ions in suppressing the growth of prostate cancer cell lines and this effect might correlate with the generation of free radicals in the culture media.^41^ Taken together, we propose that EGCG and Zn^2+^ ions released from endosomes may induce DNA damage and subsequently cell death in the cancer cells which take up the hybrid particles.

Next, fluorescence trackable doxorubicin was loaded onto the EGCG-ZnO particles and its location inside the cancer cells studied. As shown in (Fig. 5, bottom), the fluorescent image of cancer cells at 50 min post-treatment with the doxorubicin-loaded EGCG-ZnO particles showed co-localization of doxorubicin with endosomal/lysosomal compartments, indicating that the drug-loaded particles were endocytosed into cells. During this time, there was no signal from zinquin, so the particles were not yet disintegrated into Zn^2+^ ions. At 250 min post-treatment, the zinquin signals were first observed at the same location as doxorubicin and endosomal/lysosomal compartments (Fig. 5, bottom). Later, at 500 min post-treatment, the zinquin signals spread out and no longer co-localized with endosomal/lysosomal compartment signals or doxorubicin signals (Fig. 5, bottom). These results suggest that once the EGCG-ZnO particles were disintegrated, both Zn^2+^ ions and the loaded doxorubicin were released. Thus, it is likely that EGCG in the hybrid particles also leaked out from the endosomal/lysosomal compartments together with the Zn^2+^ ions.

## Conclusion

Here, hybrid EGCG-ZnO nanoparticles with 16.5 ± 5.3 nm in diameter have been successfully synthesized from ZnCl_2_ and EGCG. The EGCG-ZnO particles could kill the PC-3 human prostate cancer cells at concentrations that were not toxic to WI-38 human embryonic lung fibroblasts. Intracellular trafficking studies revealed the endocytosis of EGCG-ZnO particles into PC-3 prostate cancer cells, followed by dissolution of the particles due to the increased acidity inside the endosomal/lysosomal compartments, resulting in the release of Zn^2+^ ions along with EGCG into cytoplasm. Interestingly, we observed the accumulation of Zn^2+^ ions in nucleus, suggesting the nucleus may be the target site of Zn^2+^ for its anticancer action. It should be noted here that hybrid EGCG-ZnO particles prepared from the purchased EGCG with 96% purity also gave similar anti-cancer activity to the particles prepared from our own extracted EGCG. Anti-cancer activity of the hybrid EGCG-ZnO in animal model is under way.

## Conflicts of interest

There are no conflicts to declare.

## Supplementary Material

RA-008-C7RA10997K-s001
