# Supplementary material for: Epigallocatechin gallate-zinc oxide co-crystalline nanoparticles as an anticancer drug that is non-toxic to normal cells
Source: RSC Adv. 2018 Feb 15;8(14):7369–76. doi: 10.1039/c7ra10997k (PMC9078484; doi:10.1039/c7ra10997k)
Supplement: RA-008-C7RA10997K-s001 [file RA-008-C7RA10997K-s001.pdf]

Electronic Supplementary Material (ESI) for RSC Advances.
This journal is © The Royal Society of Chemistry 2018

Supplementary Information for

Epigallocatechin gallate-zinc oxide co-crystalline nanoparticles as anticancer drug that is non-toxic to normal cells†

Pawatsanai Samutprasert,[a,b,Ψ] Khajeelak Chiablaem,[c,Ψ] Chanon Teeraseranee,[d] Punnawich Phaiyarin,[a] Puttikorn Pukfukdee,[a] Prompong Pienpinijtham,[a] Jisnuson Svasti,[c] Tanapat Palaga,[e,f] Kriengsak Lirdprapamongkol,[c,⊥] and Supason Wanichwecharungruang*[a,f,⊥]

[a] *Department of Chemistry, Faculty of Science, Chulalongkorn University. E-mail: psupason@chula.ac.th*

[b] *Center of Excellence on Petrochemical and Materials Technology, Chulalongkorn University, Bangkok 10330, Thailand.*

[c] *Laboratory of Biochemistry, Chulabhorn Research Institute, Bangkok 10210, Thailand.*

[d] *Department of Nanoengineering, Faculty of Engineering, Chulalongkorn University.*

[e] *Department of Microbiology, Faculty of Science, Chulalongkorn University.*

[f] *Center of Excellence on Materials and Bio-interfaces, Chulalongkorn University, Bangkok 10330, Thailand.*

[Ψ,⊥] *These authors contribute equally.*

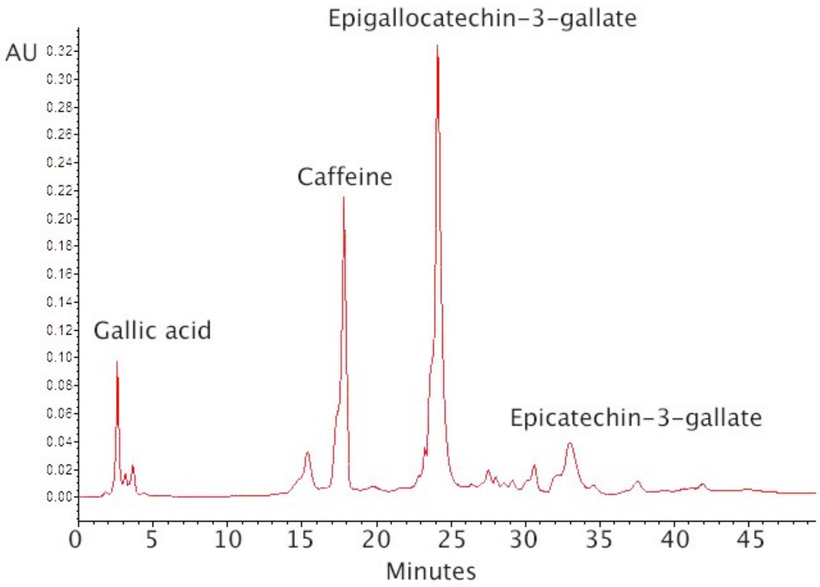

Figure S1. Chromatogram of the Oolong tea extract. The assignment of the four peaks is based on retention times of the chromatogram obtained from each standard under the same chromatographic condition.

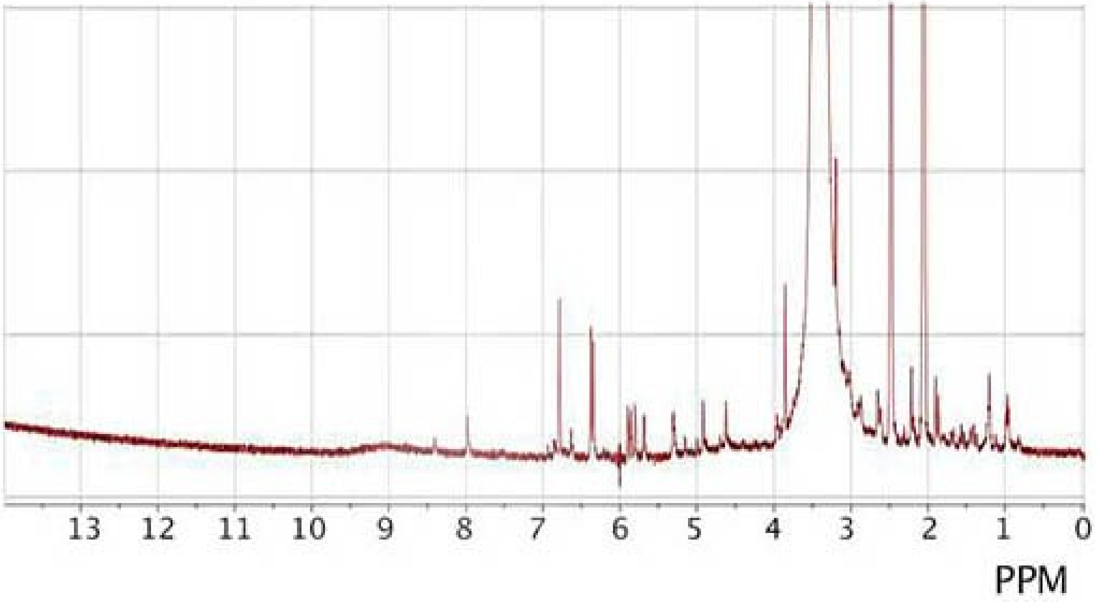

Figure S2. ^{1}H NMR spectrum of extracted epigallocatechin-3-gallate.

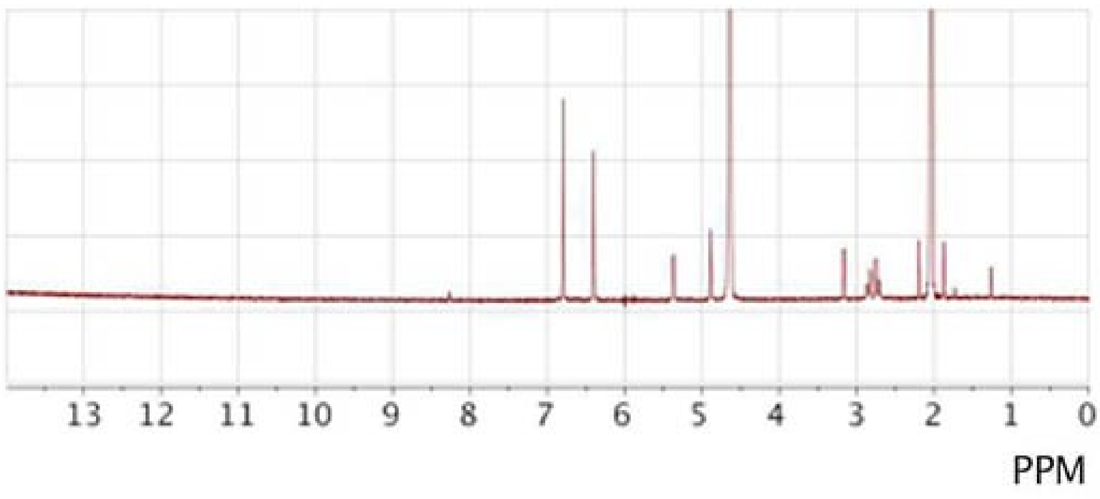

Figure S3. ^{1}H NMR spectrum of standard epigallocatechin-3-gallate.

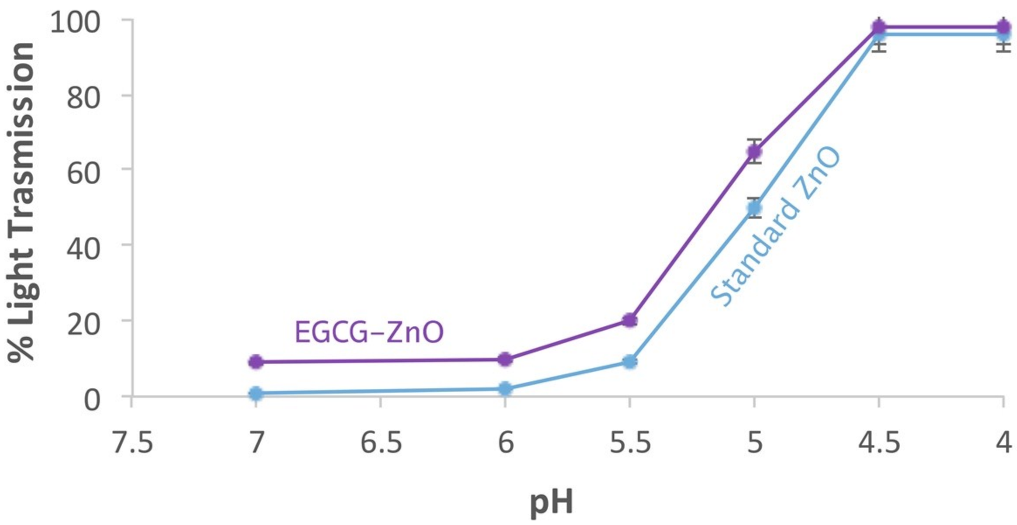

Figure S4. Degree of OTE-ZnO particle solubilization at various pH conditions in comparison to the standard ZnO, as monitored through the percentages of light transmission.

Table S1. Hydrodynamic sizes and zeta potential values of EGCG-ZnO and standard ZnO particles in water.

	Hydrodynamic sizes (nm)	Zeta potential (mV)
EGCG-ZnO	409.5 ± 9.2	-20.03 ± 0.23
Standard ZnO	297.5 ± 4.5	-25.00 ± 1.74

Table S2. IC_{50} values of EGCG-ZnO and standard ZnO particles in normal (WI-38) and cancer (PC-3) cells.

Cell types	Concentrations that causes 50% cell mortality			
	EGCG-ZnO particles		Standard ZnO particles	
	ZnO (µg/mL)	EGCG (µg/mL)	ZnO (µg/mL)	EGCG (µg/mL)
Normal	19.0	0.170	18.5	-
Cancer	10.0	0.090	17.5	-
